# Age Is Just a Number: No Impacts of Scat Ageing on Single Nucleotide Polymorphism Genotyping Using a Target Capture Approach

**DOI:** 10.1002/ece3.71755

**Published:** 2025-07-09

**Authors:** Alexis L. Levengood, Katrin Hohwieler, Daniel Powell, Romane H. Cristescu

**Affiliations:** ^1^ Detection Dogs for Conservation, School of Science, Technology, and Engineering University of the Sunshine Coast Sippy Downs Queensland Australia; ^2^ Marine and Terrestrial Megafauna Research Cluster University of the Sunshine Coast Sippy Downs Queensland Australia; ^3^ Centre for Bioinnovation University of the Sunshine Coast Sippy Downs Queensland Australia

**Keywords:** conservation genetics, DNA degradation, faecal samples, koala, low quality genetic data, non‐invasive genetic sampling, *Phascolarctos cinereus*, sample age, scat degradation, targeted genotyping

## Abstract

Recent advances in DNA sequencing and genotyping technologies are rapidly building our capacity to address ecological, evolutionary, and conservation questions for wildlife. However, wildlife genetic research increasingly relies on samples containing low quantities and quality of DNA, such as non‐invasive, archival, and environmental DNA. These samples present unique methodological challenges; for samples collected in the wild, it is important to understand the impact of environmental exposure time or sample ‘age’ on DNA quality and downstream genetic analyses. Here, we aged koala ( *Phascolarctos cinereus* ) scats under natural conditions and quantified DNA degradation. We assessed the effect of age on genetic data quality by measuring the proportion of missing single nucleotide polymorphism (SNP) data using DArTCap, a targeted genotyping methodology hypothesised to tolerate degraded DNA. Contrary to other studies, we found koala scat age was not a significant predictor of genotyping quality (i.e., rate of missing SNP calls) in the first 10 days of environmental exposure. We yielded high quality data from 10‐day‐old scats, but also low‐quality data from fresh scats. This study is the first to investigate the effect of scat age on genotyping success using a targeted approach, and DArTCap specifically. These findings support the use of targeted genotyping (such as DArTCap) from scats and provide insights for future research using DNA from non‐invasive samples. Targeted genotyping may extend the timeframe, from which accurate data can be obtained from non‐invasive samples, increasing potential sample sizes and enhancing our ability to address important questions in population ecology, conservation genetics, and population management.

## Graphical abstract

Wildlife genetic research increasingly relies on samples containing low quantities and quality of DNA, which present unique methodological challenges such as the impact of environmental exposure time or sample ‘age’ on DNA quality and downstream genetic analyses. We aged koala ( *Phascolarctos cinereus* ) scats under natural conditions and quantified DNA degradation, assessing the effect of age on genetic data quality by measuring the proportion of missing SNP data using a targeted genotyping methodology. We found koala scat age was not a significant predictor of genotyping quality (i.e., rate of missing SNP calls) in the first 10 days of environmental exposure which suggests targeted genotyping may extend the timeframe from which accurate data can be obtained from non‐invasive samples, increasing potential sample sizes and enhancing our ability to address important questions in population ecology, conservation genetics, and population management.

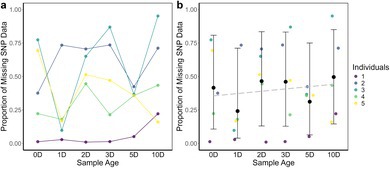

## 1 Introduction

Recent advances in molecular techniques have facilitated an increase in the use of non‐invasive genetic methods in wildlife research, management, and conservation (Deiner et al. 2017; Zemanova 2021). This is especially true for cryptic, endangered, rare, or otherwise difficult‐to‐survey species, as non‐invasive techniques allow for the sampling of wild animals without risk of causing harm or stress, while also often being safer and logistically simpler for the researcher (Deiner et al. 2017; Byrne et al. 2021; Zemanova 2021). One of the most common non‐invasive methods to obtain genetic material is the collection of scat samples, where DNA from the target animal can be isolated from intestinal wall epithelial cells exfoliated during defecation (Albaugh et al. 1992; Höss et al. 1992). Both nuclear and mitochondrial DNA from faeces are now routinely used in research and are being applied in management with the implementation of new applications (e.g., species distribution mapping, diet analysis, landscape and conservation genetics) (Waits and Paetkau 2005; Rodgers and Janečka 2013).

Although the benefits of non‐invasive genetic sampling techniques are evident (e.g., no need to capture the animal for sample collection; stress minimisation; increases the ease for sample collection from cryptic, endangered, rare, and difficult to study species), the limitations can be challenging. The major drawback of using faecal samples is the potential recovery of low quantity and quality host DNA, which can lead to high rates of genotyping errors often observed in microsatellite analyses (Taberlet et al. 1999; Morin et al. 2001; Pompanon et al. 2005) and missing data in single nucleotide polymorphism (SNP) genotyping (Pompanon et al. 2005; Schultz et al. 2018). Researchers have devoted significant effort to understanding how external factors such as environmental conditions (Piggott 2004; Murphy et al. 2007; Santini et al. 2007; Brinkman et al. 2010), diet (Murphy et al. 2007; Panasci et al. 2011), storage (Santini et al. 2007; Panasci et al. 2011), age (Piggott 2004; Santini et al. 2007; Panasci et al. 2011; Schultz et al. 2018), and often a high ratio of microbial to host DNA (Broquet et al. 2007; Ramón‐Laca et al. 2015) impact the recovery of usable DNA from faecal samples.

The age of the sample, which is the time from defecation to sample collection, is a consistent and major driver of DNA degradation in scats, regardless of species (although taxa differences can be evident). Generally, host DNA shows significant degradation within days of scats being deposited (Piggott 2004; Murphy et al. 2007; Santini et al. 2007; Panasci et al. 2011; Schultz et al. 2018). Although most research has examined the effect of age on DNA obtained from scat samples through microsatellites and polymerase chain reaction (PCR) analyses (Piggott 2004; Murphy et al. 2007; Santini et al. 2007; Panasci et al. 2011), current research is beginning to examine its impact when using SNPs (Schultz et al. 2018). Despite a surge in conservation and population genetics studies that use SNPs (Morin et al. 2004; Helyar et al. 2011; Zimmerman et al. 2020), especially those derived from DNA of non‐invasive samples such as scales (Smith et al. 2011), hair (Russello et al. 2015), and scats (Ekblom et al. 2021), as of 2025, only one published study has investigated the impacts of scat age on host DNA through SNP analyses. Schultz et al. (2018) compared the quality of genetic information of host DNA sequences generated from koala ( *Phascolarctos cinereus* ) scats that were two and 14 days old using a proprietary DArTseq protocol (from Diversity Arrays Technology (DArT), Canberra). This work highlights that the impact of scat age on host DNA using older techniques is fairly well‐studied; however, exploring this relationship through the application of SNP genotyping remains underexplored. Moreover, the relationship between scat sample age and targeted SNP genotyping, which promised better recovery of degraded DNA (Perry et al. 2010; Snyder‐Mackler et al. 2016; Hernandez‐Rodriguez et al. 2018), remains unstudied.

Although previous studies suggest that it is preferable to extract DNA from fresh faeces, typically up to 48–72 h old (Piggott 2004; Murphy et al. 2007; Santini et al. 2007; Panasci et al. 2011; Schultz et al. 2018), this may not always be feasible due to funding and time constraints. Furthermore, accurately and reliably determining scat age in the wild can be challenging, particularly the precise identification of scats less than 72 h old. Therefore, it is important to understand how sample age impacts the quality of the genotyped SNP data through next‐generation sequencing methodologies. DArTseq, a restriction enzyme complexity reduction method utilizing next‐generation sequencing platforms, has successfully yielded SNP data useful for genetic analyses, despite evidence of the effects of sample age of koala scats (Schultz et al. 2018). DArTCap, an example of targeted genotyping approaches, advances the DArTseq technology by using probes designed on specifically selected markers through an RNA–DNA method. Capture probes selectively and preferentially bind to DNA sequences flanking the targeted markers. This method produces an enriched set of DNA fragments from the target species, allowing for improved recovery from low concentration host DNA. Therefore, it is hypothesized that genotyping using DArTCap could be more tolerant of degraded DNA (Hohwieler et al. 2022).

The aim of this study was to identify possible impacts of sample age on genotype quality, using a targeted genotyping methodology (DArTCap), which could then inform sampling of faeces in the field. Here, we used scats from koalas, a cryptic, arboreal marsupial with a patchy distribution along the east coast of Australia, recently classified as endangered across much of its range (DAWE 2022). We aged koala scats through an experimental setup by exposing them to natural environmental conditions over the course of 10 days to determine how scat age affects SNP genotyping using targeted methodologies (DArTCap).

## 2 Methods

Fresh scats were collected from five temporarily housed wild adult koalas (two females, three males) that were generally in good health from areas around Southeast Queensland (Oliveros et al. 2023). Scats were collected from the ground of each individual koala's enclosure in the morning; all enclosures had been cleaned the night before. Six pellets were collected from each individual. Scats were then placed in sterile tubes covered with a layer of gauze to allow the escape of volatile compounds (Oliveros et al. 2023). Tubes were transferred to a research facility and kept at ambient temperature and protected from direct sunlight. One scat from each koala was frozen on arrival (−20°C). Based on the time at which koala cages were cleaned, these scats were less than 12 h old (named 0D in results).

To simulate the effect of natural environmental degradation processes on scats over time (scat age), the remaining five scats from each koala were placed on individual toothpicks embedded into five Styrofoam boards. To simulate natural tree and ground cover conditions, Styrofoam boards with scats were then placed within a mesh enclosure and located near the base of a tree in a remnant forest patch. This setup ensured scat exposure to natural fluctuations in temperature, light, rainfall, and wind, but provided protection from insects/animals, as shown in Oliveros et al. (2023) Supporting Information. Scats were aged under these conditions over a 10‐day period (from 20/11/2020–29/11/2020). Weather data from this period can be seen in Table S1 and were consistent with weather conditions of this season in southeast Queensland. It is also worth noting that rainfall is known to wash away target DNA from scats in the wild across many species, and as such, samples are not collected for most species post rainfall events (Piggott 2004; Brinkman et al. 2010; Oehm et al. 2011; Panasci et al. 2011; Wedrowicz et al. 2013; Agetsuma‐Yanagihara et al. 2017; McInnes et al. 2017); therefore, the limited rainfall present in this study accurately represents natural field sampling conditions adhered to in most research settings.

Scats were sampled for DNA extraction at six time points: 0 (< 12 h old), 1, 2, 3, 5, and 10 days (*N* = 30 scats, six scats per five individual koalas). At each time point, one scat per individual was removed from the outdoor setup and transferred to the laboratory and frozen at −20°C. Samples from time point zero were those scats placed in the freezer directly after collection and not exposed to the environmental conditions. All scats were stored frozen (Piggott and Taylor 2003) to maintain consistency with common laboratory protocols and to reflect the real‐life impracticality of extracting scats on the same day as field collection. After all scats were collected at their respective time points, they were stored frozen for 5.5 months before DNA extraction. Currently, there are no published data on the effect of freezing time on the quality of DNA extracted from koala scats. Additionally, no unscheduled freeze/thaw events occurred during this time, and analyses post‐freezing were undertaken to be consistent with most current research methodologies.

### 2.1 DNA Isolation From Scats

All DNA extractions were performed by a single researcher to ensure that inter‐individual differences in extraction performance did not unintentionally influence the results of downstream analyses. The exfoliated koala intestinal epithelial cells present on the surface of the scat were targeted by slicing off and retaining the outer‐most layer of the scat using a sterile scalpel. DNA was then extracted from these outer surface slices using the QIAmp PowerFecal Pro DNA kit (Qiagen), following the manufacturer's protocol, with the following variations: after adding CD1 buffer, samples were incubated at 65°C for 1 h, and then vortexed for 7 min at maximum speed using a Genie 2 Vortex Mixer (Scientific Industries). Final DNA isolates were eluted in 100 μL of C6 elution buffer and concentrated down to a volume of 50–60 μL using Eppendorf's Vacufuge Vacuum Concentrator (Eppendorf Inc., Germany). DNA quantity was measured in nucleic acid (ng/μL) count, using a Thermo‐Scientific Nanodrop 2000 Spectrometer, while quality was assessed via gel electrophoresis using a 1% agarose gel containing ethidium bromide using 5 μL of extracted scat DNA (See Figure S1). Purified DNA was stored at −80°C.

### 2.2 β‐Actin qPCR

To compare koala DNA content between samples and gain a better understanding of DNA input versus genotyping data quality, a koala β‐actin assay was performed (Shojima et al. 2013). The β‐actin quantitative real‐time PCR (qPCR) assay was run using the iTaq Universal SYBR Green Supermix (Bio‐Rad) on a CFX 96 Touch System (Bio‐Rad). The concentrations of all DNA samples were standardized to 40 ng/μL prior to the assay. The β‐actin qPCR assay was compared to a standard curve generated from a dilution series of PCR products of a known concentration (10^6^–10^1^). The reaction mixture contained 5 μL of DNA template, 10 μL of SYBR Green Supermix, 0.5 μL of 0.25 μM β‐actin forward primer, and 0.25 μM reverse primer, and 4 μL of nuclease‐free water to make up to a final volume of 20 μL. Primers used for koala β‐actin gene were 5′‐GAGACCTTCAACACCCCAGC‐3′ (forward) and 5′‐GTGGGTCACACCATCACCAG‐3′ (reverse) designed to amplify a 111 bp segment of koala β‐actin DNA (Shojima et al. 2013). DNA samples were run in duplicate to check repeatability. The absence of contamination by exogenous DNA was verified by the inclusion of a negative control containing all reagents but no sample DNA. Reactions were performed with an initial denaturing of 95°C for 3 min, followed by 40 cycles of denaturation at 94°C for 15 s, annealing at 60°C for 30 s, extension at 72°C for 30 s, and data acquisition at 82°C for 10 s, followed by melt curve analysis from 65°C to 95°C in 0.5°C increments. Results were processed using Bio‐Rad CFX Manager software, and the melt curve showed melting temperature was consistent across all samples. Koala DNA was measured via the quantification cycle (Cq) in the β‐actin assay. Cq values are inverse to the amount of target genetic material in the sample, and therefore lower Cq values indicate higher target DNA content. As such, lower counts of β‐actin, and thus less koala DNA, correspond to higher Cq scores.

### 2.3 SNP Genotyping

One DNA sample for each individual from each sampling time point was used for SNP genotyping. SNP genotyping was conducted by Diversity Arrays Technology, Canberra, using DArTCap technology. DArTCap involves a combination of complexity reduction methods and next‐generation sequencing platforms. We utilized koala‐specific capture probes (designed using DNA obtained from koalas in Southeast Queensland) that bind to restriction fragments in the representations carrying the specific DArTseq markers, aiming for approximately 2000 SNP markers. All five koalas came from populations, from which the capture probes were designed. Further details regarding SNP genotyping for non‐invasive koala scat through DArTCap can be found in Hohwieler et al. (2022).

### 2.4 Analyses

The proportion of missing data (i.e., SNPs that were not called during genotyping) was calculated for all scat ages of each individual (*N* = 30), after the removal of monomorphic loci. We further investigated average SNP read coverage for each sample as an additional measure of data quality. We ran a binomial generalized linear mixed model (GLMM) using the *lme4* package (Bates et al. 2014) in R version 4.1.1 (R Development Core Team 2010) to determine whether sample age impacted the proportion of missing data in SNP results, when including individual ID as a random effect.

## 3 Results

**FIGURE 1:**
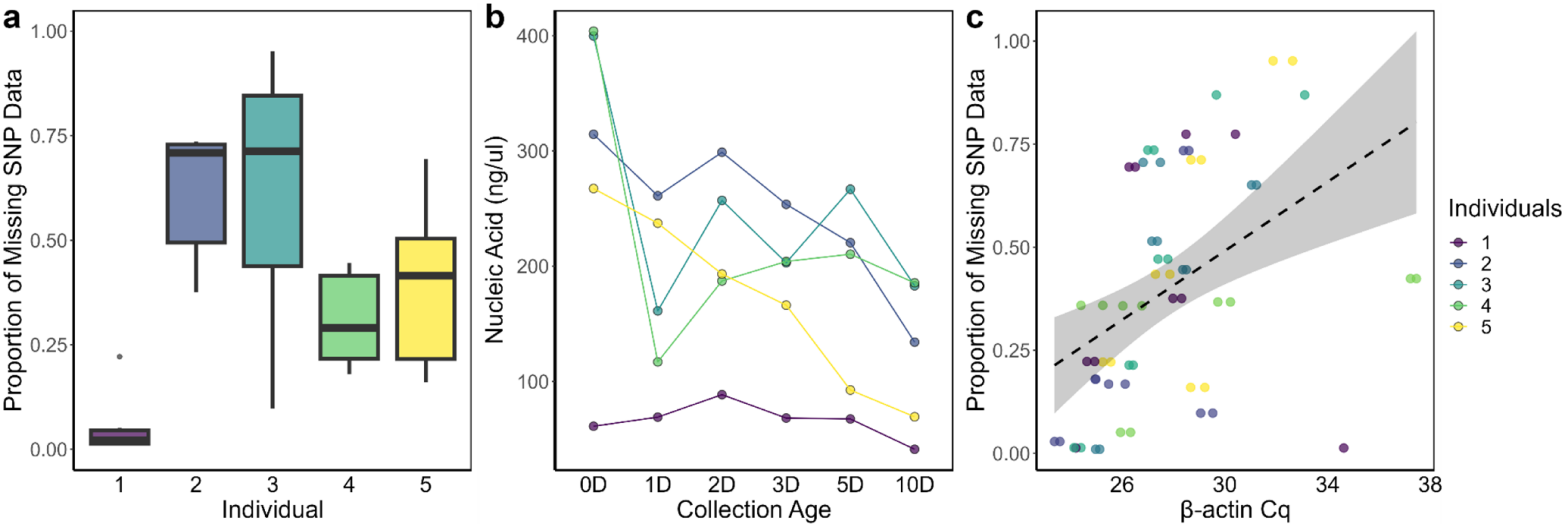
Individual variation in genetic quality. (a) The proportion of missing SNP data (across all ages) and (b) nucleic acid (ng/uL) count from koala scat DNA extractions. Each individual (*n* = 5) denoted by a different colour. D = days. (c) The relationship between β‐Actin Cq scores (done in duplicate) and the proportion of missing SNP data with a linear regression line. A Cq of 28.4 is approximately equivalent to 1000 gene copies per μL.

All samples were genotyped and used for further analyses. Individual samples varied in both their proportion of missing SNP data (range = 16%–96%) and the total nucleic acid yield (range = 41–404 ng/μL, Figure 1a,b). As expected, the average read coverage per SNP had a negative relationship with the proportion of missing SNP data, reflecting the quality of the genetic sample (Figure S2). High nucleic acid yields (Figure 1b), as measured by Nanodrop, did not equate to high genetic data quality (i.e., low missing SNP data, Figure 1a); interestingly, the opposite trend was present (Pearson's Correlation coefficient *R* ^2^ = 0.541, *p* < 0.01). β‐actin, however, did show a relationship with genetic quality, where lower counts of β‐actin per sample (e.g., higher Cq scores) resulted in greater SNP missingness (Figure 1c, Pearson's Correlation coefficient *R* ^2^ = 0.479, *p* < 0.01) and lower average read coverage per SNP (Figure S3), for example, samples with low amounts of koala DNA performed more poorly in SNP genotyping. Additionally, the concentration of β‐actin gene copies varied between individuals (Table S2, Figure S4) and might be linked to genotyping quality. For example, the concentration of β‐actin gene copies from individual 1 ranged between 918.3 (day 5) and 4091.6 (day 1), with an average of 2292.8 gene copies per μL of DNA; 2.5–25‐fold greater than the other individuals. Pending further replication, we might infer that a concentration of approximately 1000 gene copies of β‐actin per microliter of extracted DNA is required to generate a dataset comprised of the most accurate SNP calls observed in our study (of less than 15% missing SNP data).

**FIGURE 2:**
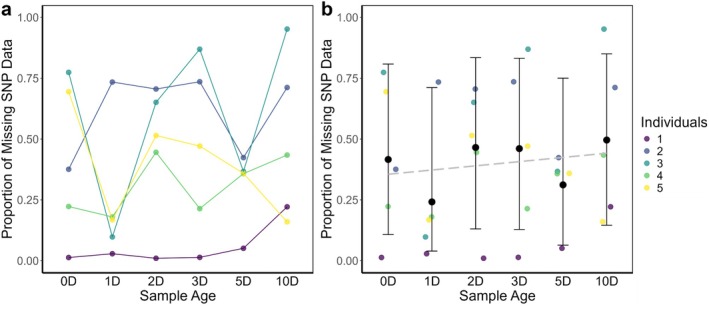
Impacts of koala scat age on genetic data quality. (a) The proportion of missing SNP data from DNA obtained from koala scats aged (i.e., exposed to the natural elements) across six time points and (b) binomial generalized linear mixed model (GLMM) results with the proportion of missing SNP data from extracted aged scats including 95% CIs. Raw data (from graph a) included on graph b. The grey dashed line represents the average linear trend. *n* = 5 individuals, D = days.

In addition to individual variation in missing SNP data, the proportion of missing SNP call data varied with the age of the scat sample (Figure 2a,b). Although not consistent across all individuals, visually there was a slight trend showing that the proportion of missing SNP data increased with scat age (Figure 2a,b). However, when accounting for individual identity, scat age was not a significant predictor of the proportion of missing data from koala SNP DNA, and we noted high variability (large 95% CI overlaps) between ages (*p*‐values > 0.05, Table 1, Figure 2b, Table S3, Figure S5). In other words, no scat age (i.e., time point) yielded better quality data (i.e., low missing SNP data) than any other (Table 1, Figure 2, Table S3, Figure S5). The lack of an effect of age is further evidenced by no trends observed in β‐actin scores across age (Figure S6).

**TABLE 1:** Results of the effect of age on the proportion of missing SNP data.

Parameter	Estimate	SE	*z*	*p*	AIC
Intercept	−0.562	1.200	−0.468	0.640	47.1
Age – 1D	−1.232	1.624	−0.759	0.448	
Age – 2D	2.357	1.735	1.359	0.174	
Age – 3D	1.111	1.540	0.722	0.471	
Age – 5D	−1.232	1.624	−0.759	0.448	
Age – 10D	1.111	1.540	0.722	0.471	

*Note:* Results from a binomial generalised linear mixed model (GLMM) with individual ID as the random effect. Intercept represents time point zero (zero days from excretion). *n* = 5 individuals.

## 4 Discussion

Our results showed that, between zero and 10 days, the age of a koala scat sample had no significant influence on genotyping quality using a targeted genotyping methodology. Specifically, we found that older samples (up to 10 days old) can yield higher quality genetic data than previously assumed, based on previous findings from a non‐targeted approach (Schultz et al. 2018). Unfortunately, we also confirmed that, as one might expect from non‐invasive samples, even very fresh samples (collected immediately after defecation, with no time for environmental degradation) can yield poor genetic data quality. These results highlight the variable quality and quantity of genetic material and data that can be obtained from non‐invasive samples, as well as the power of targeted next‐generation genetic approaches, such as DArTCap. By enriching target DNA (Perry et al. 2010; Hernandez‐Rodriguez et al. 2018) that usually constitutes less than 5% of the total DNA obtained from a faecal sample (Perry et al. 2010; Snyder‐Mackler et al. 2016), targeted approaches may minimize the impact of time and environmental degradation on faecal DNA utility. Based on non‐invasive samples from wild koalas, these findings suggest that a larger window post‐defecation may be open for ecologists to collect samples in the wild than previously thought (i.e., up to 48–72 h in (Schultz et al. 2018)), which could translate into larger sample sizes when in the field.

Our results further support that total nucleic acid yield from scat DNA extraction is not a good indicator of the quality of genotyping results (Hayward et al. 2022), which we confirmed here for koala samples. This result is unsurprising, as high nucleic acid yield from non‐invasive samples, in contrast with DNA isolated from tissue or blood samples, is often indicative of noise from non‐target DNA such as environmental fungi, substrate material, diet material, and host microflora (bacterial, viral, and parasitic) in koalas (Peterson et al. 2009; Schultz et al. 2018; Littleford‐Colquhoun et al. 2022; Oliveros et al. 2023) and other animals (Taberlet et al. 1999; McInnes et al. 2017). β‐actin gene quantification was found to be a much better indicator of downstream genetic data quality, demonstrating that samples with low koala DNA composition (i.e., low ratio of koala DNA compared to other, non‐target DNA) generally performed poorly in SNP genotyping. The relationship between koala DNA content present in the extracted sample and the ability to maximise the number of SNP calls recovered could help establish data filtering thresholds and highlights the potential for β‐actin assays to serve as quality control monitors of DNA extraction quality. This marker utility would likely vary between species and may require optimisation; however, it could assist in standardising input prior to library preparation and establishing minimum thresholds for host DNA content.

Contrary to other studies with similar scat aging times (i.e., DArTSeq SNP analyses of koala scat: 14 days [Schultz et al. 2018], microsatellite analyses of coyote: 5 days [Panasci et al. 2011], brush‐tailed rock‐wallaby and red foxes: 2 to 3 days [Piggott 2004], wolf: 3 days [Santini et al. 2007] and brown bears: 3 days [Murphy et al. 2007]), we found that, up to 10 days between defecation and scat collection, the quality of SNP data did not decrease. Our results indicated that DNA extracts obtained from 10‐day‐old scat samples subjected to the conditions imposed at our study site were just as informative as samples collected at day zero. However, our results confirm that fresh samples (e.g., day zero) did not always yield high‐quality DNA and that some fresh samples resulted in poor genetic data quality. This high variation in the quality of genetic data is interesting and needs further investigation. For example, we are yet to know how fecal DNA varies per scat in a defecation event (i.e., does the first scat excreted in a defecation event garner more DNA through an increase in exfoliated epithelial cells?) (Walker et al. 2009) and/or how much/how consistent individuals vary in DNA shed through defecation. Results from our sample size of five indicate high variability between individuals, yet why or how consistent that variability is remains unanswered. We therefore suggest that sampling and extracting multiple scats per individual would be best practice to ensure good DNA recovery.

When using DArTCap methodologies on DNA obtained from koala scats, our results suggest there is no significant relationship between genetic quality and sample age, up to 10 days post‐defecation. This lack of a relationship could be explained by multiple, non‐mutually exclusive possibilities. Firstly, the lack of impact of sample age could simply be an artefact of a small sample size. However, this is unlikely as previous studies using SNP analyses with comparable sample sizes have identified significant effects of age on DNA degradation (Schultz et al. 2018). A second possible explanation is that the timeframe used here was too short in the conditions we tested (though we chose a similar timeframe to other degradation studies [Santini et al. 2007, Panasci et al. 2011; Agetsuma‐Yanagihara et al. 2017; Schultz et al. 2018]), and degradation may occur slowly, over a longer time period. Degradation over time has not been investigated using targeted genotyping and may impact results more slowly than observed in other studies using non‐targeted approaches (e.g., Piggott 2004; Murphy et al. 2007; Santini et al. 2007; Panasci et al. 2011; Schultz et al. 2018). Further research over longer sample ageing time periods (beyond 10 days) should be explored to determine when genetic quality decreases using targeted genotyping. Alternatively, other external factors (e.g., variation in diet material, or shed cells/DNA present on each scat, the presence of residual cleaning products on enclosure floors) may have influenced our results, masking or confounding any impact of age, a possibility that is difficult to control for or test post hoc. Finally, DArTCap may show greater resilience in data recovery, with less impact of sample age or the effects of low target DNA quantity and quality. This suggests that DArTCap and other targeted genotyping approaches may provide a more robust genotyping methodology for successful genotyping from a broader range of field‐collected scats (though additional testing should occur). The likelihood of this is supported by the advancements of DArTCap over DArTSeq technologies, particularly its use of a probe design that improves the capture and sequencing of low‐concentration DNA, making it better suited for degraded DNA (Hohwieler et al. 2022).

Our results suggest that, while some DNA degradation occurs up to and likely beyond 10 days post‐defecation, the level of degradation within this timeframe is not sufficient to affect data quality, as measured by missing SNP calls. Although previous studies suggest that extracting DNA from fresh scats is preferable, with significant degradation typically occurring within 48–72 h (Piggott 2004, Murphy et al. 2007, Santini et al. 2007, Panasci et al. 2011, Schultz et al. 2018), we surmise that the DArTCap methodology will enable collection of samples up to 10 days post‐defecation without scat age significantly impacting data quality. However, other factors should not be overlooked and, whether due to improved methods or untested drivers of data quality, our results highlight the importance of strategic field sampling to enable the collection of a greater number and variety of samples.

This study revealed considerable variation in host DNA recovery between individuals, in terms of quantity and quality. While the reasons for this variation remain unclear, it is unlikely to be due to storage methods or DNA extraction protocols, which were consistent throughout the study. It is likely that individual factors such as diet or the amount of host cellular material on scats may play a role. Volatile compounds and phenolics produced from the koala diet, which can vary between individuals depending on the plant species consumed, are known to cause cell membrane damage and accelerated DNA degradation (Khan and Hadi 1998; Carson et al. 2006). Additionally, differences in the amount of epithelial cellular material shed on the surface of scats can also vary and may further influence DNA recovery (Albaugh et al. 1992; Kohn and Wayne 1997).

Finally, although our results suggest that time since defecation did not affect genetic quality when left under natural conditions outdoors for up to 10 days and analyzed using DArTCap methodologies, it is important to note that for this study, scats were placed on the ends of toothpicks, avoiding direct contact with the ground. Further, they were protected from insects and other organisms, potentially limiting exposure to decomposers (Santini et al. 2007). Variable environmental factors such as rainfall, ground cover conditions, or ultraviolet light exposure can influence DNA degradation and impact DNA recovery (Piggott 2004; Murphy et al. 2007; Brinkman et al. 2010; Cristescu et al. 2012). Hence, although no age effects were found in our study, the collection of aged scats from the ground or under different environmental conditions may produce different results. As such, future research focused on the effects of environmental drivers of target DNA degradation in scats using target capture approaches (as has been done using traditional PCR approaches) may be useful to further assess the utility of these advanced methodologies and assessing impacts under controlled conditions may provide further insights moving forward.

## 5 Conclusion

Understanding the influence of sample age on targeted genotyping success is important for balancing trade‐offs in field and laboratory methodologies. Our findings suggest that using a targeted approach can allow for greater flexibility by enabling the use of older scat samples without significant downstream impacts on analysis. For koalas, this means targeted genotyping approaches could maximize sample collection efforts, increasing sample sizes to address key questions in population ecology, conservation genetics, and population management. Though our results are specific to koalas, further research investigating the effects of sample age using targeted next generation genotyping methodologies may demonstrate their utility for other species. This could be particularly valuable for genetic studies of cryptic, evasive, endangered, or otherwise difficult to study species, as older samples previously considered non‐viable may yield valuable data using these advanced technologies.

## Author Contributions

**Alexis L. Levengood:** formal analysis (lead), investigation (lead), methodology (supporting), visualization (lead), writing – original draft (lead). **Katrin Hohwieler:** data curation (lead), formal analysis (supporting), writing – review and editing (supporting). **Daniel Powell:** formal analysis (supporting), investigation (supporting), methodology (supporting), writing – review and editing (supporting). **Romane H. Cristescu:** conceptualization (lead), funding acquisition (lead), investigation (supporting), methodology (supporting), supervision (lead), writing – review and editing (supporting).

## Conflicts of Interest

The authors declare no conflicts of interest.

## Supporting information


Data S1.

## Data Availability

Data is available at https://doi.org/10.5061/dryad.n5tb2rc7h.
