# Supplementary material for: Age Is Just a Number: No Impacts of Scat Ageing on Single Nucleotide Polymorphism Genotyping Using a Target Capture Approach
Source: Ecol Evol. 2025 Jul 9;15(7):e71755. doi: 10.1002/ece3.71755 (PMC12240678; doi:10.1002/ece3.71755)
Supplement: Supplementary file 1 — Data S1. [file ECE3-15-e71755-s001.docx]

**Title:** Age is just a number: No impacts of scat aging on SNP genotyping using a target capture approach

**Authors:** Alexis L. Levengood^1,2^, Katrin Hohwieler^1,2^, Daniel Powel^1,3^, Romane H. Cristecu^1,2*^

**Affiliations:**

1. Detection Dogs for Conservation, School of Science, Technology, and Engineering, University of the Sunshine Coast, Sippy Downs QLD, 4556, Australia
2. Marine and Terrestrial Megafauna Research Cluster, University of the Sunshine Coast, Sippy Downs QLD, 4556, Australia
3. Centre for Bioinnovation, University of the Sunshine Coast, Sippy Downs QLD, 4556, Australia

***Correspondence to:** [rcristes@usc.edu.au](mailto:rcristes@usc.edu.au)

| Date | Temperature High  (°C) | Temperature Low  (°C) | Max wind  (km/h) | Max Humidity  (%) | Max Barometer  (mbar) | Precipitation  (mm) | Solar exposure  (MJ m^2^) |
| --- | --- | --- | --- | --- | --- | --- | --- |
| 20/11/20 | 26.8 | 18.8 | 18 | 81 | 1021 | 0 | 29.6 |
| 21/11/20 | 27.4 | 16.7 | 17 | 93 | 1021 | 0 | 30.0 |
| 22/11/20 | 27.5 | 18.0 | 17 | 90 | 1019 | 0 | 30.4 |
| 23/11/20 | 28.0 | 17.5 | 33 | 93 | 1015 | 0 | 30.3 |
| 24/11/20 | 30.3 | 20.2 | 23 | 90 | 1009 | 0 | 25.6 |
| 25/11/20 | 26.2 | 23.6 | 26 | 82 | 1013 | 5 | 7.1 |
| 26/11/20 | 27.7 | 18.6 | 19 | 80 | 1014 | 0 | 30.3 |
| 27/11/20 | 27.8 | 18.4 | 15 | 90 | 1018 | 0 | 29.9 |
| 28/11/20 | 27.8 | 18.3 | 22 | 80 | 1017 | 0 | 30.5 |
| 29/11/20 | 28.7 | 17.9 | 35 | 91 | 1014 | 0 | 30.7 |

**Table S1:** Sunshine Coast weather for November 20-29^th^ 2020. Historical weather for the Sippy Downs area (Queensland) based on the Bureau of Meteorology weather station located at the Sunshine Coast airport (located 13.4 km away).


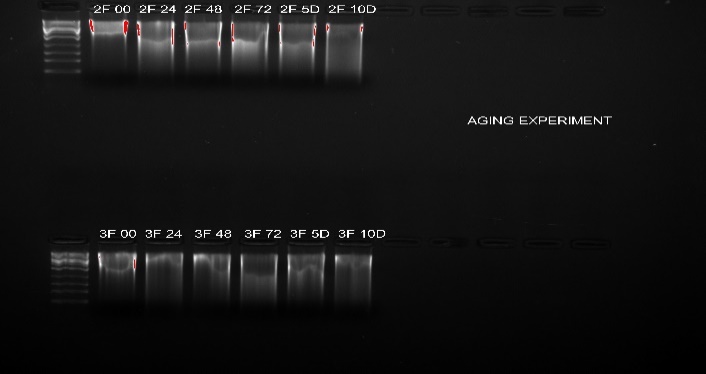


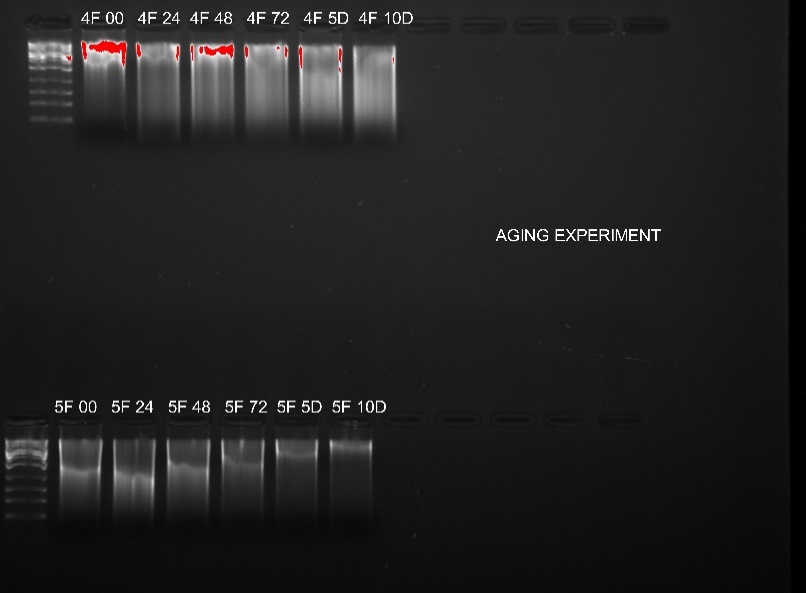


0D 1D 2D 3D 5D 10D

Sample

4


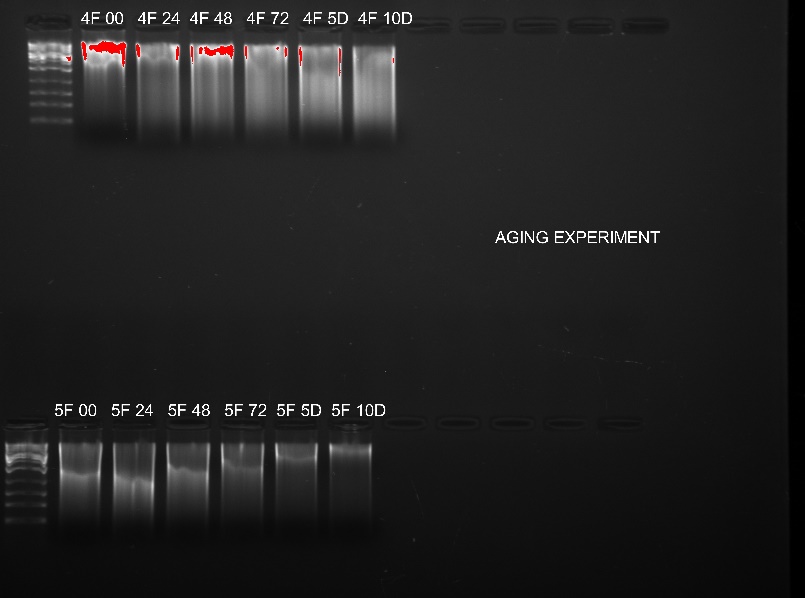


0D 1D 2D 3D 5D 10D

Sample

5

Sample 2: 0D 1D 2D 3D 5D 10D

Sample 3: 0D 1D 2D 3D 5D 10D


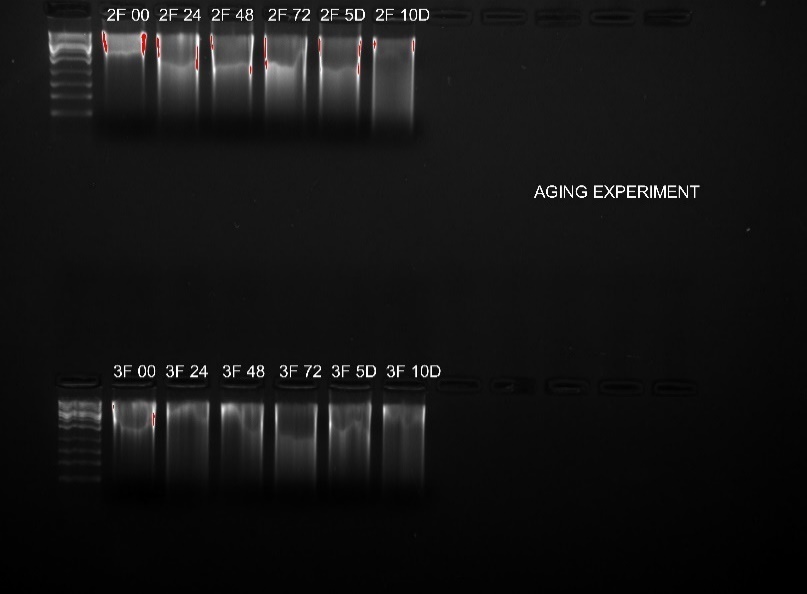

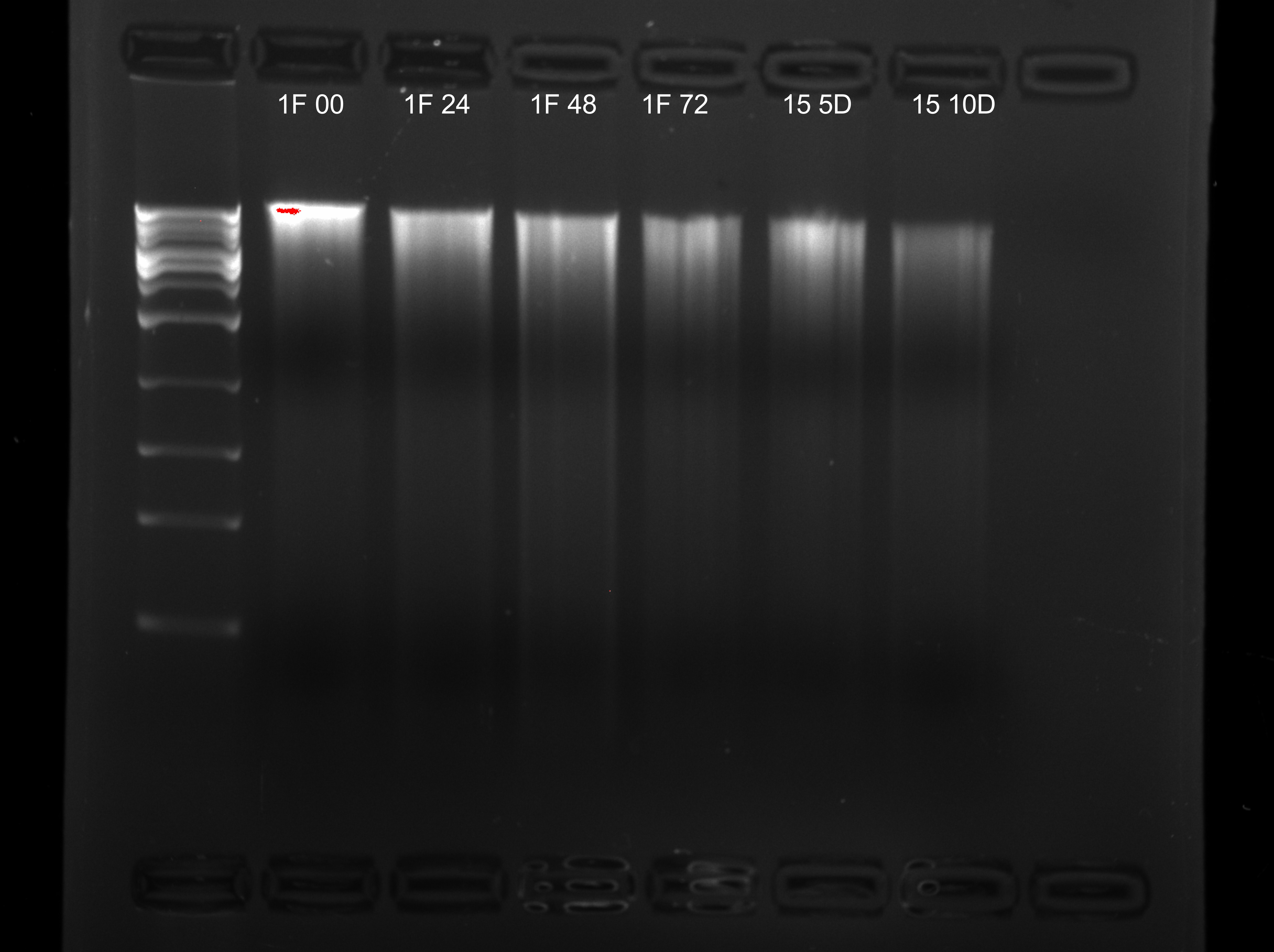

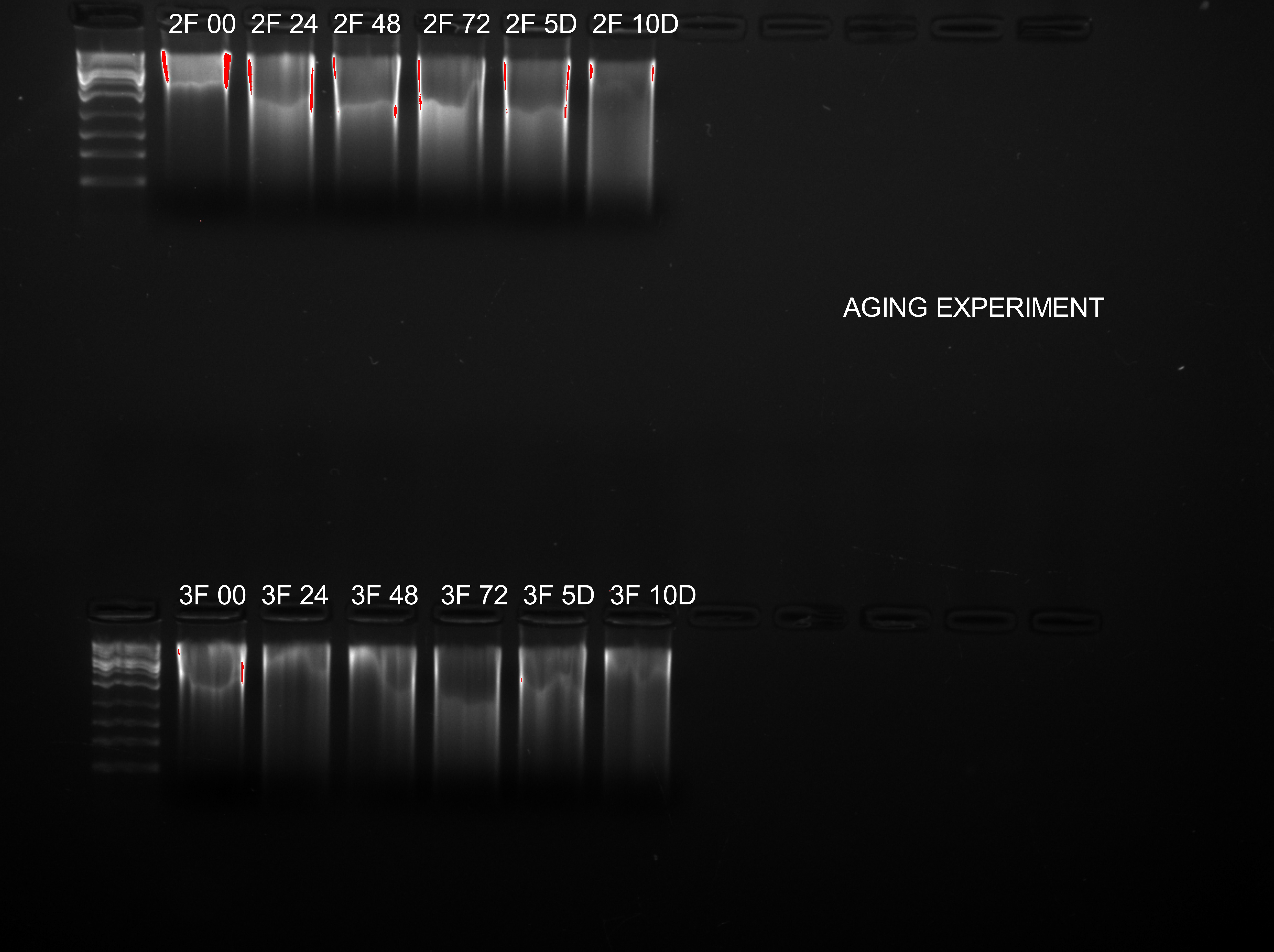

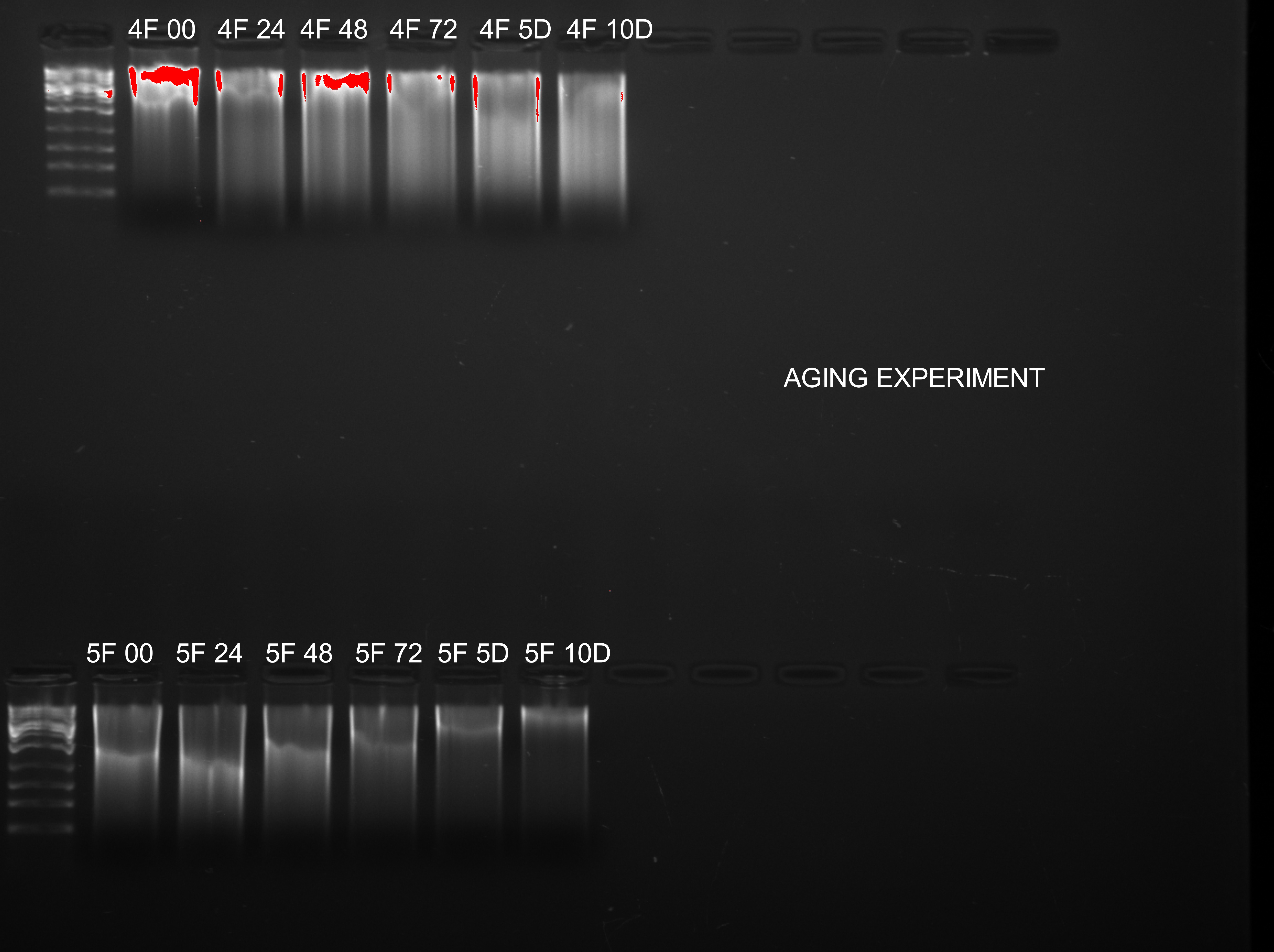


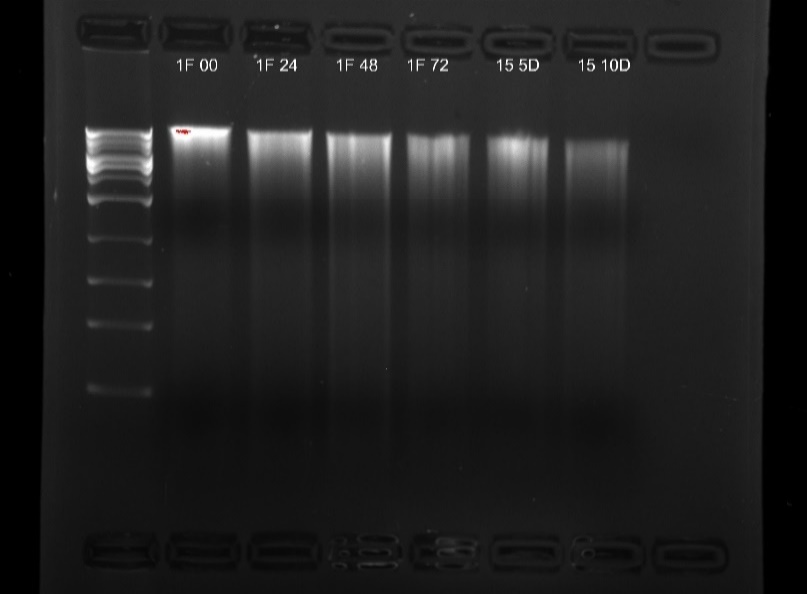


Sample 1: 0D 1D 2D 3D 5D 10D

**Figure S1:** Gel electrophoresis image results for each individual koala (n=5) at the six time points (0D, 1D, 2D, 3D, 5D, 10D). D = days. Brightness of band indicates high quantity of DNA. A larger smear (i.e., downward spread) with a lower band indicates more degraded DNA.

**
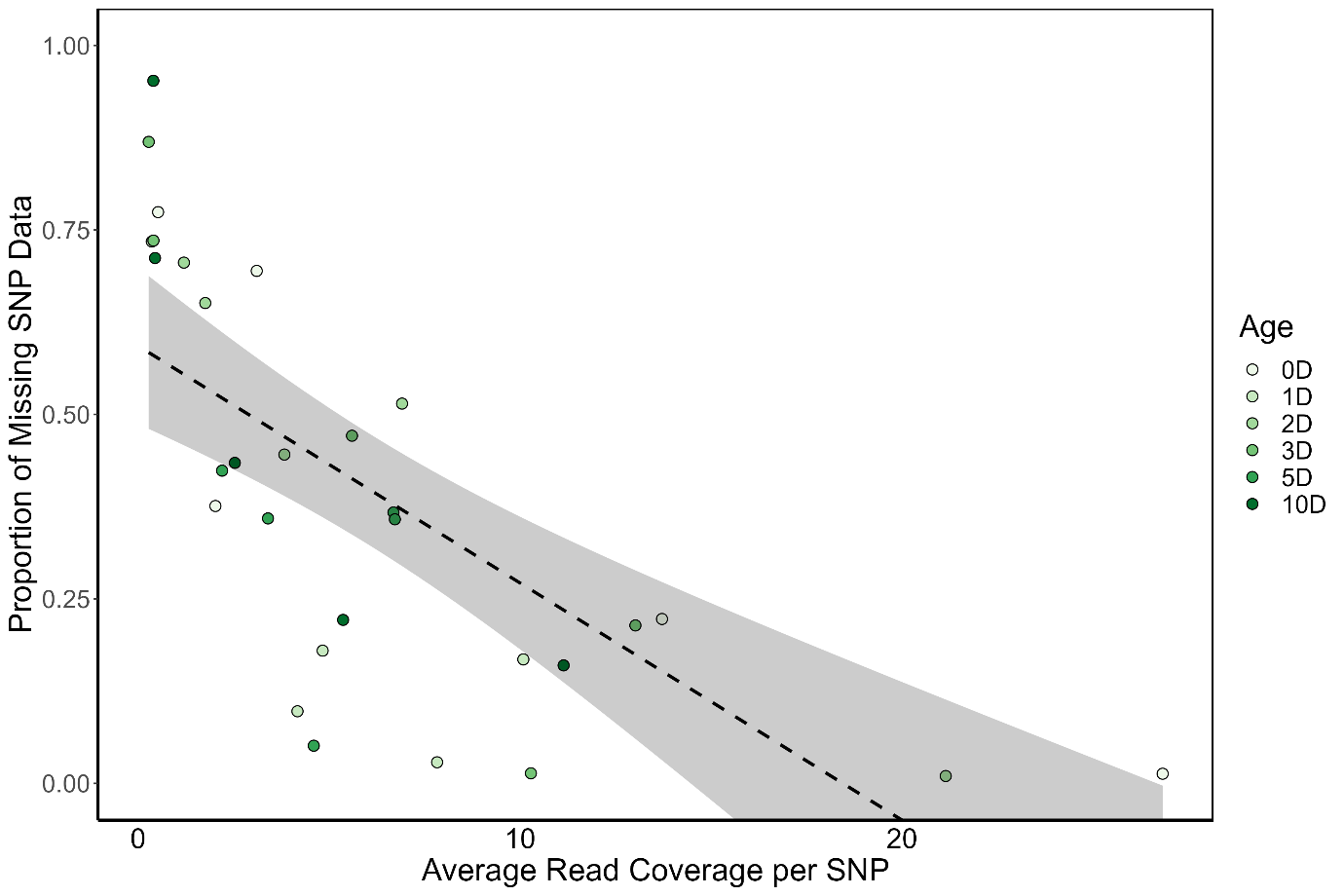
**

**Figure S2:** The proportion of missing SNP data from extracted koala DNA of scats aged (i.e., exposed to the natural elements) against the average read coverage per SNP from genotyped data. n = 5 individuals across six time points resulting in 30 samples total.

**
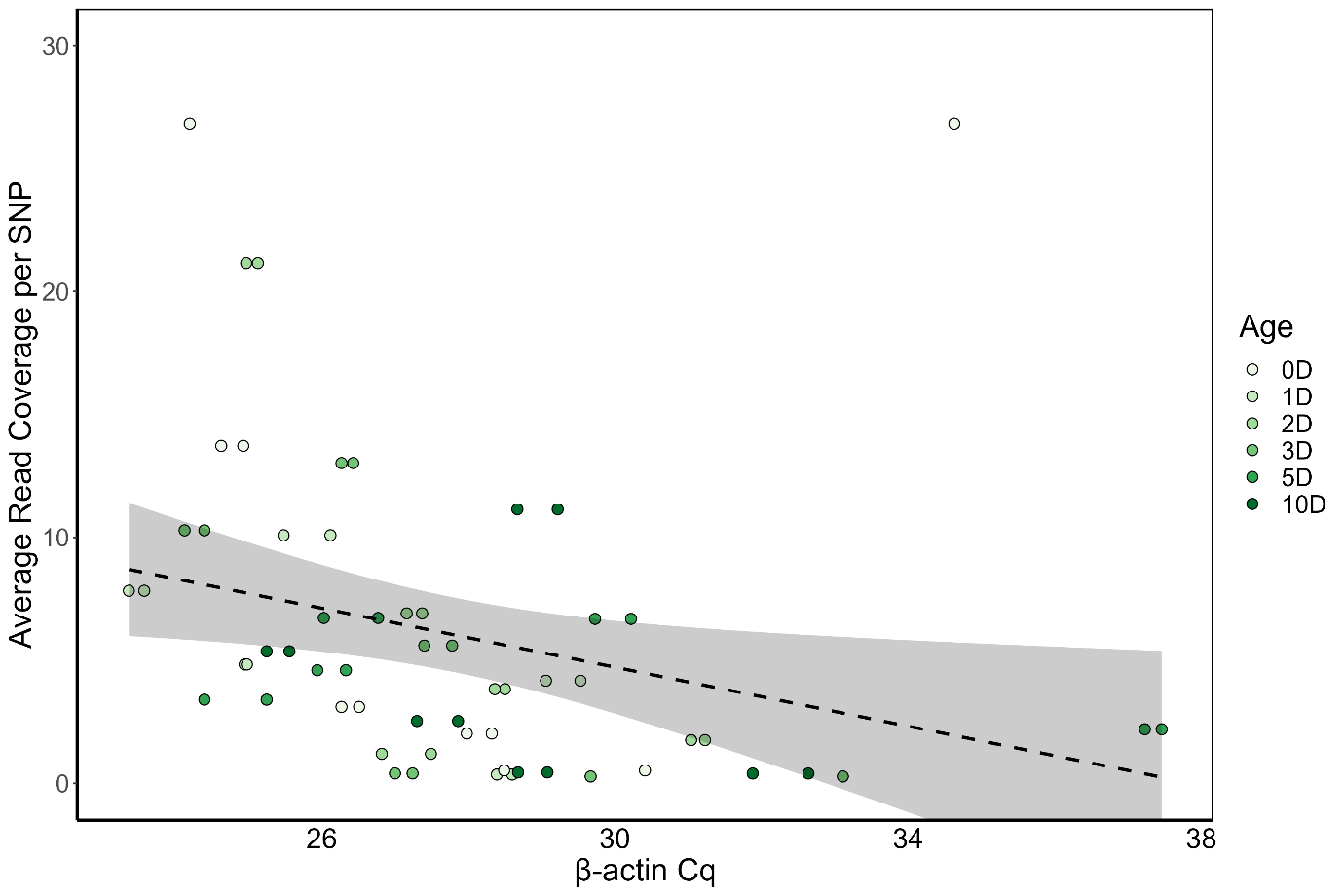
**

**Figure S3:** The linear regression of average read coverage per SNP from genotyped data against β-actin Cq scores (done in duplicate) of five koala scat samples aged across six time points (n = 30 samples total).

**Table S2:** Average β-actin gene copies per µl found in koala scats per individual koala.

| Individual Koala | Average β-actin gene copies |
| --- | --- |
| 1 | 2,292.8 |
| 2 | 220.1 |
| 3 | 89.1 |
| 4 | 924.7 |
| 5 | 816.8 |

**
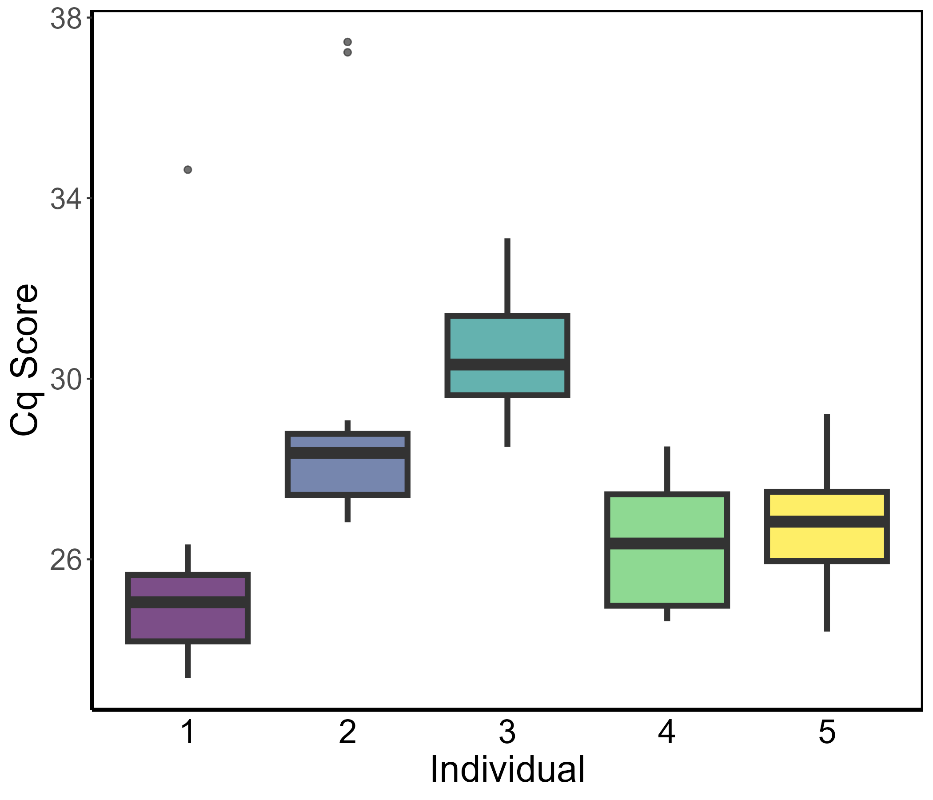
**

**Figure S4:** Variation in β-actin gene copies (Cq scores) from koala scat DNA extractions per individual koala (n = 5).

**Table S3:** Pairwise comparison using Generalised Linear Hypothesis Testing (GLHT) with Tukey contrasts controlling for multiple testing for the investigation of age effects on the proportion of missing SNP data. D = days.

| Parameter | Estimate | SE | z-value | p-value |
| --- | --- | --- | --- | --- |
| 0D – 1D | -1.351 | 1.721 | -0.785 | 0.963 |
| 0D – 2D | 1.115 | 1.537 | 0.725 | 0.974 |
| 0D – 3D | 0.000 | 1.543 | 0.000 | 1.000 |
| 0D – 5D | -1.135 | 1.006 | -0.113 | 1.000 |
| 0D – 10D | 0.000 | 1.543 | 0.000 | 1.000 |
| 1D – 2D | 2.466 | 1.790 | 1.378 | 0.702 |
| 1D – 3D | 1.351 | 1.721 | 0.785 | 0.963 |
| 1D – 5D | -1.000 | 1.006 | -0.099 | 1.000 |
| 1D – 10D | -1.351 | 1.721 | -0.785 | 0.963 |
| 2D – 3D | -1.115 | 1.537 | -0.725 | 0.974 |
| 2D – 5D | -1.247 | 1.006 | -0.124 | 1.000 |
| 2D – 10D | 1.115 | 1.537 | 0.725 | 0.974 |
| 3D – 5D | 1.135 | 1.006 | 0.113 | 1.000 |
| 3D – 10D | 0.000 | 1.543 | 0.000 | 1.000 |
| 5D – 10D | -1.135 | 1.006 | -0.113 | 1.000 |


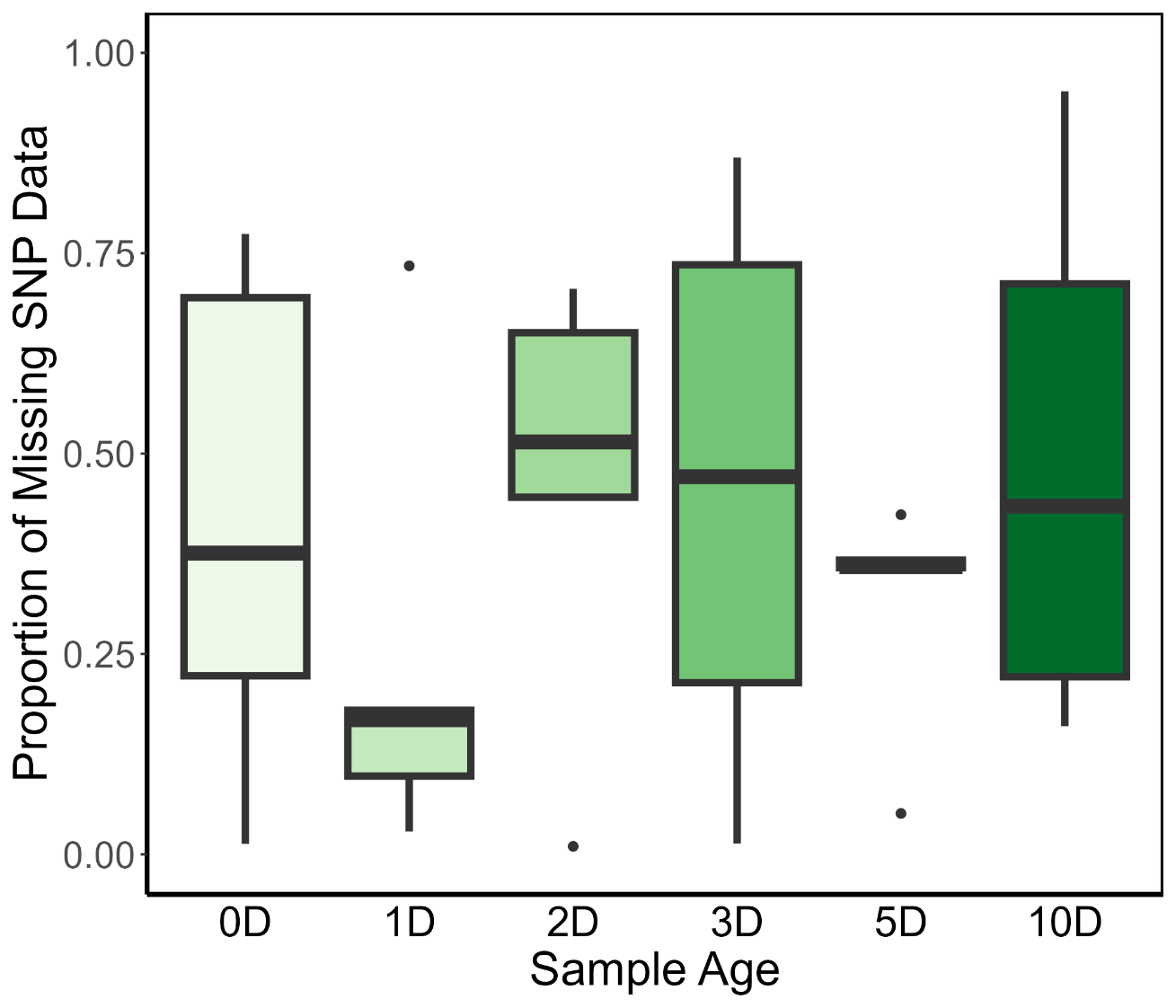


**Figure S5:** The proportion of missing SNP data from DNA extracted koala scats aged (i.e., exposed to the natural elements) and sampled across six time points. n = 5 individuals, 30 scats total, D = days.


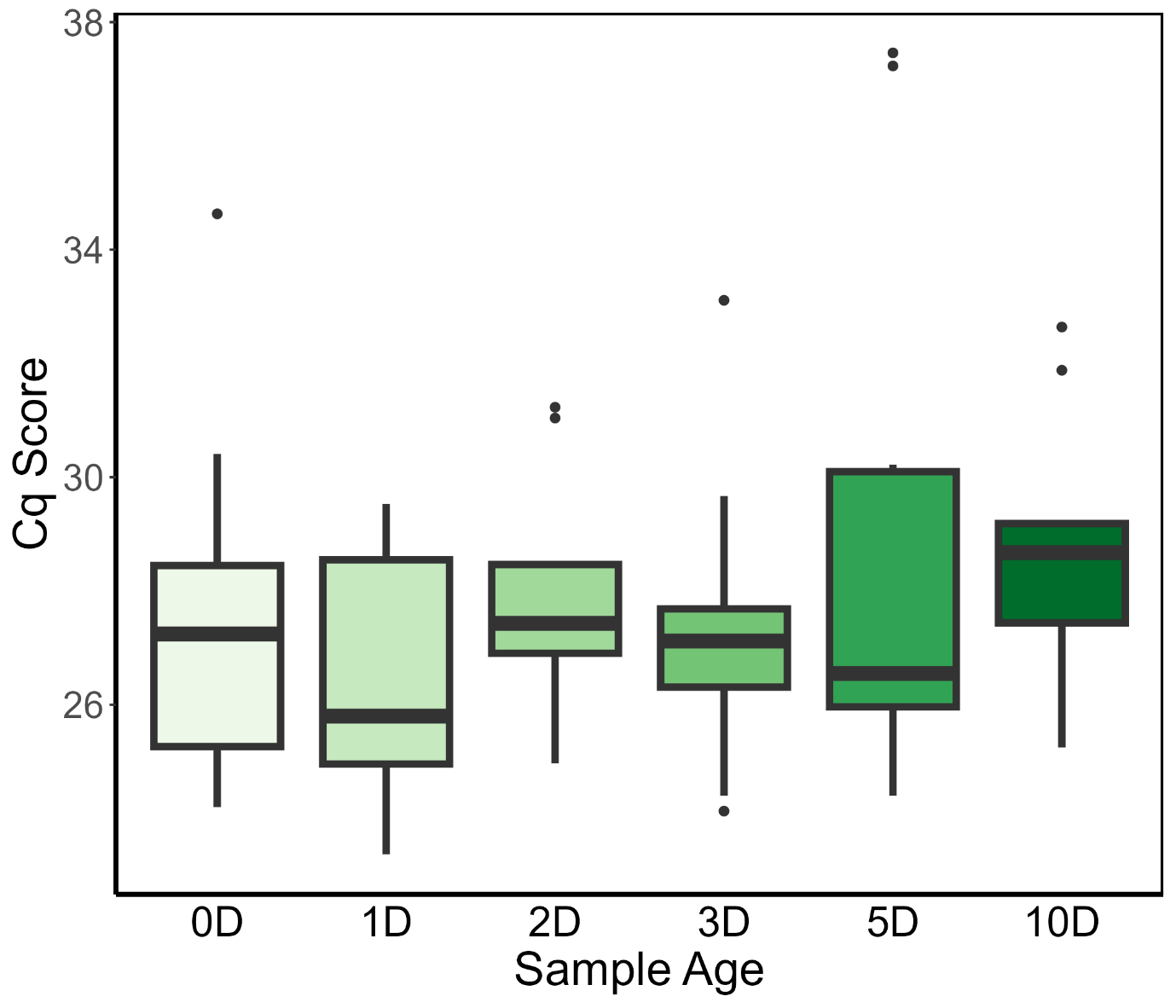


**Figure S6:** β-actin gene copies (Cq scores) from koala scat DNA extractions where scats were aged (i.e., exposed to the natural elements) and sampled across six time points. n = 5 individuals, 30 scats total, D = days.
